# A Syringe-Like Love Dart Injects Male Accessory Gland Products in a Tropical Hermaphrodite

**DOI:** 10.1371/journal.pone.0069968

**Published:** 2013-07-24

**Authors:** Joris M. Koene, Thor-Seng Liew, Kora Montagne-Wajer, Menno Schilthuizen

**Affiliations:** 1 Animal Ecology, Department of Ecological Science, Faculty of Earth and Life Sciences, VU University, Amsterdam, The Netherlands; 2 Terrestrial Zoology, Naturalis Biodiversity Center, Leiden, The Netherlands; 3 Institute for Tropical Biology and Conservation, Universiti Malaysia Sabah, Kota Kinabalu, Sabah, Malaysia; 4 Institute Biology Leiden, Leiden University, Leiden, The Netherlands; Institut Pluridisciplinaire Hubert Curien, France

## Abstract

Sexual conflict shapes the evolution of many behaviours and processes involved in reproduction. Nearly all evidence supporting this comes from species where the sexes are separated. However, a substantial proportion of animals and most plants are hermaphroditic, and theoretical work predicts that sexual conflict plays an important role even when the sexes are joined within one individual. This seems to have resulted in bizarre mating systems, sophisticated sperm packaging and complex reproductive morphologies. By far the best-known example of such a strategy in hermaphrodites is the shooting of so-called love-darts in land snails. All known love darts carry a gland product on their outside and enter this into the partner’s hemolymph by stabbing. Here, we show that species of the snail genus *Everettia* possess a syringe-like dart that serves as a real injection needle. Their dart is round in cross-section, contains numerous channels, and has perforations along its side. Histology and electron microscopy show that these holes connect to the channels inside the dart and run all the way up to the elaborate mucus glands that are attached to the dart sac. This is the first report on a love dart that is used as a syringe to directly inject the gland product into the partner’s hemolymph. Although the exact use and function of this dart remains to be demonstrated, this clearly adds to the complexity of the evolution of reproductive strategies in hermaphrodites in general. Moreover, the perforations on the outside of the love dart resemble features of other injection devices, thus uncovering common design and repeated evolution of such features in animals.

## Introduction

Sexual selection favours traits that enhance reproductive success. Darwin [1] phrased sexual selection mainly in a pre-copulatory context to explain extravagant male secondary sexual characteristics. Parker [2] argued that sexual selection could also act post-copulatorily whenever sperm compete for the fertilisation of eggs, and termed this sperm competition. By extending pre- and post-copulatory sexual selection theory, research has firmly established that sexual encounters are usually accompanied by conflicts of interest between partners [3], [4]. Such sexual conflicts arise because traits that are adaptive for one sex can be detrimental to the other. As a result, these conflicts can trigger co-evolutionary arms races leading to costly and sometimes bizarre mating behaviours [5]–[7]. For species with separate sexes, many recent studies have focused on sperm competition, sexual conflicts, counter-adaptive arms races and their evolutionary consequences [3]. Many of these investigations have shown that conflicts between the sexes can have important implications for the evolution of (secondary) sexual characteristics and behaviours [8].

For simultaneous hermaphrodites, although the theoretical framework already exists and predicts a vital role for sexual selection and conflict [9]–[11], the importance of pre- and post-copulatory sexual selection and sexual conflict for the evolution of mating systems and reproductive morphologies has been addressed only relatively recently [12]–[14]. Especially during the past decade, experimental evidence in support of sexual conflict in hermaphrodites has accumulated [15]–[18]. Nevertheless, sexual selection in hermaphrodites is fundamentally different from the situation in separate sexes, because within each mating a simultaneous hermaphrodite can gain fitness through its male as well as its female function, and often has the additional option to fertilise itself [19]. Indeed, theoretical modelling indicates that this situation may lead to the evolution of even more harmful reproductive traits in hermaphroditic species than in species with separate sexes [9], [20], [21]. The latter is consistent with the bizarre mating systems, sperm packaging, and reproductive morphologies found in hermaphrodites [4], [12]. Examples of this include hypodermic insemination in tropical flatworms [22], partner sedation in sea slugs [23], hypodermic injection of gland products in earthworms and sea slugs [18], [24] and highly complex sperm design in flatworms [25].

By far the best known example of such a strategy is the shooting of so-called love-darts in land snails [3], [4], [26]. Such darts, which are usually calcareous, are forcefully stabbed through the partner’s skin during courtship or copulation. For the species *Cornu aspersum*, it has been demonstrated that the penetration of the dart through the partner’s body wall transfers a gland product into the recipient’s hemolymph [27]. This gland product has been found to cause conformational changes in the female reproductive system, which impede the entrance to the sperm digesting organ (bursa copulatrix) [28], [29]. Following this discovery, a series of subsequent studies investigating sperm storage and paternity ultimately led to a very elegant experiment which convincingly confirmed that the mucus on the dart is indeed responsible for the increased paternity of the shooter [30]. Whereas the sperm donor benefits from shooting a love dart, the dart receiver may be disadvantaged because its skin is injured, infection risk may be increased, and the process of sperm storage is altered [31], [32]. Evidence for this resulting in a conflict between the two mating partners comes from an inter-species comparison, which provided clear support for antagonistic co-evolution between love-darts and spermatophore-receiving organs [16].

Although the abovementioned correlational study provided the first support for the predicted co-evolutionary arms races in simultaneous hermaphrodites in response to sexual conflict, all direct evidence hinges on findings obtained for the garden snail *C. aspersum*. This species has a dart that is bladed and shot once into the partner, where it stays behind in the skin. However, love darts come in all kinds of shapes and sizes with some species possessing more than one [16]. Moreover, darts are used in different ways during mating. For example, rather than stabbing a disposable dart once, some species stab repeatedly with a reusable dart [33], [34]. In order to explore the extremes of this variation, we here investigated *Everettia corrugata corrugata* Laidlaw 1937, a simultaneously hermaphroditic tropical land snail species that is endemic to Mount Kinabalu, Sabah (Malaysia) and belongs to the family Dyakiidae.

## Results and Discussion

**Figure 1:**
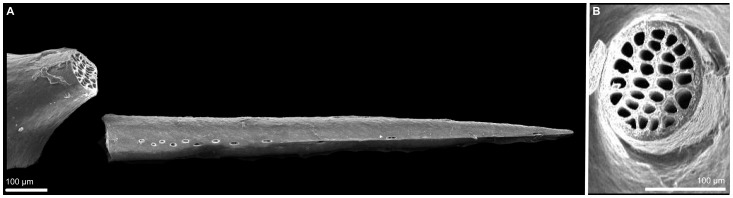
Scanning electron micrograph of the love dart of *Everettia corrugata corrugata*. **A.** The side view illustrates the row of perforations on one of the two sides. **B.** The cross section shows the channels inside the shaft of the dart that connect to the perforations along the dart’s flank.

As can be seen in the electron micrographs in Figure 1, the dart of *Everettia corrugata* has no clear blades protruding from its surface, although it does flatten towards the tip as many darts do. However, it does have two features that are unique in terms of love dart morphology. Firstly, the dart has two rows of perforations along its length, on two opposing sides (only one row can be seen in Figure 1A) [35]. Secondly, as the cross section clearly illustrates (Figure 1B), rather than being a hollow shaft, the dart’s inside is divided into many channels. Towards the tip, the number of channels gradually becomes smaller. This clearly indicates that each channel running inside the dart connects to one of the perforations along the dart’s side.

**Figure 2:**
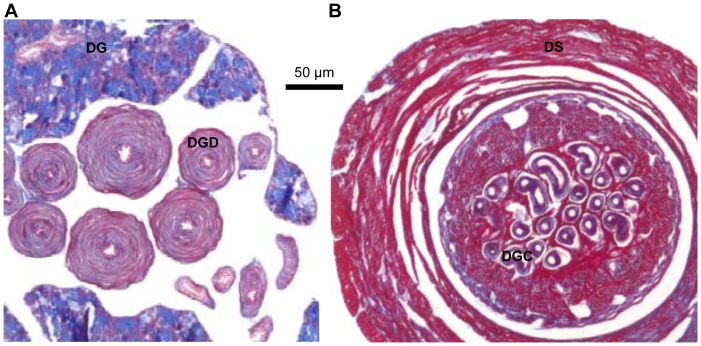
Histological sections of the dart gland and sac. **A.** The section of the dart gland shows the muscular dart-gland ducts (DGD) surrounded by dart-gland tissue (DG, dart-glands). **B.** The section of the dart sac (DS) shows its thick muscular layer. Inside the sac, many of the dart-gland channels (DGC) leading into the channels of the dart can be clearly seen. These sections are taken from the same individual, but note that the dart in Figure 1 is from a different individual.

Our histological work further illustrates the implications of this dart’s morphology for its use during copulation. As already known from morphological descriptions [36], the glands of the dart sac are attached at its posterior end via ducts that feed into the dart sac. These ducts and some of the glandular tissue are shown in Figure 2A. Based on the staining used, the glands clearly have several different types of secretory cells. Each duct, in which the secretions of the gland are collected and transported, has a thick layer of muscle cells surrounding its lumen. These muscular ducts can be followed all the way into the dart sac and the channels of the love dart. Figure 2B illustrates the latter and also shows that the wall of the dart sac is mainly made up of muscle, as is known to be the case for other species [16].

Taken together, the perforations in the dart itself as well as the co-evolved muscular lining of the gland ducts and channels inside the dart are clearly indicative of active injection. So far, stylommatophoran love darts were only known to carry the active substance(s) on their outside and enter this into the partner’s hemolymph by stabbing [16], [30], in some cases repeatedly [33], [34]. Our findings uncover the first example of a species where the dart appears to serve as a true injection needle. It now remains to be established how this dart is used during mating, but given its intricate attachment to the dart sac and gland ducts, we expect this dart is retained by the shooter after use and reused in a next mating. Its unique way of injecting, together with the fact that this species is distantly related to the other land snail species in which love darts have been studied, clearly adds to our understanding of the evolution of this bizarre reproductive attribute in hermaphroditic snails. Moreover, they highlight the importance of looking beyond model species in order to come to a detailed understanding of the evolution of bizarre accessory reproductive structures used during mating.

Finally, these findings also add to the understanding of injection devices in nature and culture. Side-perforations are, for instance, found in marinade injectors and anaesthetics needles to prevent clogging of the needle tip and to spread the injected product [37]. Designs that prevent clogging in nature are found in the venom apparatus of predatory conoidean snails that have an adapical opening for hypodermic delivery [38], [39] and snake fangs that have evolved a venom canal [40]. These general patterns in design indicate that side-perforations are one solution to optimize injection of a substance and might further inspire the field of biomimicry in the same way as the mosquito proboscis did [41].

## Methods

For this study, nine specimens of *Everettia corrugata corrugata* were collected between 2005 and 2009 at Laban Rata station (6°3′31″N 116°33′59″E, *ca*. 3100 m elevation), Mount Kinabalu, Sabah, Malaysia. The animals were collected under research permit TS/PTD/5/4/Jld. 35 (57), granted by Sabah Parks to TSL. The voucher specimens were kept in the BORNEENSIS collection, Universiti Malaysia Sabah. Different specimens were used for the electron microscopical and histological investigation.

For electron microscopy, the four darts of four individuals was carefully dissected out from the dart sac. For cross-sections, darts were carefully broken in two. The intact and broken darts were then placed on small aluminium plates with an electrically conducting adhesive, coated with platinum, and placed under a scanning electron microscope (JSM 5610, JEOL) to take pictures.

For histology, five animals were fixed in Bouin’s solution O/N and subsequently transferred to 70% ethanol. The dart sac and attached glands were then dissected out. These were dehydrated in ascending alcohol concentrations, after which the organs were transferred from 1∶1 ethanol:amylacetate, via 100% amylacetate and 1∶1 amylacetate-paraffin to be embedded in paraffin for serial sectioning at 7 *µ*m. The sections were stained with haematoxylin and eosin.
